# Right Anterior Thoracotomy Versus Partial Sternotomy for Isolated Aortic Valve Replacement: A Propensity Analysis of Clinical Outcomes and Hospital Costs

**DOI:** 10.3390/medicina62050856

**Published:** 2026-04-30

**Authors:** Massimo Baudo, Serge Sicouri, Mikiko Senzai, Yoshiyuki Yamashita, Francesco Cabrucci, Dimitrios E. Magouliotis, Farah Mahmud, Thomas Capista, Scott M. Goldman, Basel Ramlawi

**Affiliations:** 1 Department of Cardiac Surgery Research, Lankenau Institute for Medical Research, Main Line Health, Wynnewood, PA 19096, USA; sicouris@mlhs.org (S.S.); mkksnx@gmail.com (M.S.); yoshiyuki.yamashita.md@gmail.com (Y.Y.); francesco.cabrucci.6@gmail.com (F.C.); dmagouliotis@gmail.com (D.E.M.); ramlawib@mlhs.org (B.R.); 2 Department of Cardiac Surgery, Lankenau Heart Institute, Main Line Health, Wynnewood, PA 19096, USA; fm826@nyu.edu (F.M.); capistat@mlhs.org (T.C.); goldmans@mlhs.org (S.M.G.)

**Keywords:** cardiac surgery, minimally invasive, aortic valve, hospital cost, SAVR

## Abstract

*Background and Objectives*: Previous comparisons between right anterior mini-thoracotomy (RAT) and partial upper sternotomy (PS) for aortic valve replacement (AVR) have shown similar clinical outcomes. This study aims to assess the potential in-hospital cost differences in one technique over the other. *Materials and Methods*: Between 2018 and 2023, 303 patients at our institution underwent minimally invasive isolated AVR (241 PS vs. 62 RAT). Endocarditis, emergencies, and reinterventions were excluded. A 1:1 nearest neighbor propensity-matched analysis without replacement was performed. Perioperative clinical outcomes and hospital costs were analyzed, comparing total and average (per patient) direct, indirect, and total hospital costs between the two groups. Multivariable linear regression identified significant predictors of hospital costs. *Results*: Sixty-two well-matched pairs were analyzed. Significant differences were found in intraoperative (PS: 27/62, 43.5% vs. RAT: 10/62, 16.1%, *p* = 0.002) and postoperative transfusions (PS: 33/62, 53.2% vs. RAT: 16/62, 25.8%, *p* = 0.003), and median intensive care unit (ICU) hours (PS: 52.2 vs. RAT: 45.7, *p* = 0.007). Average direct, indirect, and total hospital costs were significantly higher for PS (*p* = 0.038, *p* = 0.040, and *p* = 0.035, respectively), with significant blood bank cost differences favoring RAT (*p* = 0.010). Multivariable linear regression showed that intraoperative and postoperative transfusions, ICU, and hospital length of stay were significantly associated with hospital costs, but not the surgical approach. *Conclusions*: PS and RAT have comparable perioperative clinical outcomes, with differences observed only in the number of transfusions and ICU stay, both favoring RAT. Given the significant perioperative differences and regression analysis results, the cost advantage of RAT is likely mediated through its impact on these perioperative outcomes.

## 1. Introduction

Minimally invasive surgical aortic valve replacement (SAVR) has emerged as an alternative to conventional median sternotomy (MS) for isolated aortic valve replacement, offering potential benefits such as reduced perioperative morbidity, shorter hospital stays, and improved patient satisfaction [1,2]. The two most widely adopted minimally invasive SAVR approaches are partial upper sternotomy (PS) and right anterolateral mini-thoracotomy (RAT). The PS technique typically involves making a small incision below the sternal angle, followed by an oscillating J-shaped cut extending to the third or fourth intercostal space. Surgeons may opt for alternative incision patterns, such as T, L, or inverted V shapes [3]. In contrast, the RAT approach is generally performed via an incision at the second or third intercostal space. However, RAT can present greater technical challenges compared to MS due to its more confined surgical field and the deeper position of the valve [4]. While PS has been the most commonly performed technique, RAT, which preserves the sternum entirely, has been proposed as a potentially superior alternative due to its association with lower ventilation times, reduced transfusion requirements, and less postoperative pain [5,6]. RAT has been validated across primary isolated AVR for both aortic stenosis and regurgitation, redo operations on previously sternotomized patients with hostile or high-risk mediastinal anatomy, and patients at elevated risk of poor sternal healing. Appropriate patient selection also depends on favorable anatomy [7].

Despite these advantages, there is still no consensus on the preferred minimally invasive approach. Moreover, a lack of randomized studies directly comparing PS and RAT underscores the need for high-quality evidence to guide surgical decision-making in minimally invasive SAVR. While previous studies have examined the clinical and surgical outcomes of minimally invasive SAVR [5,6,8,9,10], the associated costs have been rarely reported in the literature [11,12].

This study aims to evaluate the perioperative clinical outcomes as well as the potential in-hospital cost differences in one technique compared to the other, using a propensity-score matched population.

## 2. Materials and Methods

This retrospective study included patients who underwent minimally invasive isolated SAVR via either RAT or PS for severe native valve AS at our institution, between January 2018 and December 2023. The objective was to compare perioperative clinical outcomes and hospital costs between RAT and PS. The study protocol received approval from our Institutional Review Board, and the requirement for individual patient consent was waived due to the retrospective design. Clinical and procedural data were extracted from medical records, and all patients underwent preoperative transthoracic and/or transesophageal echocardiography before the procedure. Transfusions consisted of any blood product (i.e., red blood cells, fresh frozen plasma, cryoprecipitates, and platelets). The STS/SCA/AmSECT/SABM clinical practice guidelines on blood management were followed [13].

The study included patients with severe native valve AS, defined by an aortic valve area of ≤1.0 cm^2^ (or an aortic valve area index of ≤0.6 cm^2^/m^2^), a mean gradient of ≥40 mmHg, or a peak aortic valve velocity of ≥4.0 m/s, as assessed by transthoracic echocardiography at rest. Patients were excluded if they had a history of prior aortic valve intervention, if the procedure was primarily indicated for aortic regurgitation or endocarditis, if they underwent concomitant procedures (including aortic root enlargement), or if the procedure was performed on an emergency basis.

The surgeons performing SAVR using the RAT approach had previously achieved proficiency with this technique prior to joining our institution. Therefore, no learning curve occurred during the timeframe analyzed. Likewise, all surgeons performing PS, including those experienced in RAT, had already surpassed their respective learning curves before the start of the study period. The choice between techniques applied only to cases operated on by surgeons performing RAT. Van Praet et al. proposed a CT-based categorization of the spatial relationship between the ascending aorta and the sternum, identifying progressive leftward deviation as unfavorable for this approach [14]. However, in our experience few patients are deemed unsuitable for RAT, as minor adjunctive exposure maneuvers generally allow adequate access, but RAT is not used when concomitant procedures are required, whereas PS may still accommodate selected additional interventions.

The primary endpoint of this study aims to assess the potential in-hospital cost advantages of one technique over the other. Clinical and surgical outcomes and their relationship with hospital costs were also evaluated.

### 2.1. Surgical Technique

In the RAT group, a right femoral cut-down was performed to identify the femoral artery and vein, followed a right anterior thoracotomy over the third intercostal space with rib shingling and soft tissue retraction. Cardiopulmonary bypass (CPB) was established via femoral cannulation, and myocardial protection was achieved with cardioplegia in the ascending aorta. Following valve replacement, the aortotomy was closed, de-airing was performed, and the patient was weaned from CPB. The rib was reconstructed using a titanium plate and screws.

In the PS group, an upper partial sternotomy was performed. CPB was established via direct ascending aortic cannulation and right femoral venous cannulation. Myocardial protection was achieved with cardioplegia delivered to the aortic root. Following valve replacement and aortotomy closure, thorough de-airing maneuvers were performed, the cross-clamp was removed, and the patient was weaned from CPB.

Decannulation and heparin reversal with protamine were performed routinely in both groups, and prosthesis function was confirmed by intraoperative echocardiography.

### 2.2. Hospital Costs

Total hospital cost was defined as the aggregate of direct and indirect costs. Direct costs included all patient-care–related expenses incurred during the index admission. The category “surgical other” included non-implant disposable materials utilized during the operative and anesthetic phases. Indirect costs reflected institutional overhead and ancillary support services, including administrative operations, health information management, information technology, facility maintenance, human resources, financial services, and regional administrative functions. Four financial costs (2 per group) were not retrievable by the finance team. In these patients, clinical outcomes were available and were not excluded from the clinical analysis.

### 2.3. Statistical Analysis

Categorical variables were expressed as frequencies and percentages, with group comparisons performed using either the Chi-square test or Fisher’s exact test, depending on the data distribution. The normality of continuous variables was assessed using the Kolmogorov–Smirnov test. Normally distributed variables were reported as means with standard deviations and compared using Student’s *t*-test. Non-normally distributed variables were presented as medians with interquartile ranges (IQR) and analyzed using the Mann–Whitney U test.

To ensure comparability between groups, propensity score matching (PSM) was performed in a 1:1 ratio using the nearest-neighbor method without replacement. Matching variables included age, sex, body mass index, hypertension, diabetes, STS-PROM, NYHA class III-IV, ejection fraction, smoking status, chronic obstructive pulmonary disease, urgency, atrial fibrillation, liver disease, and prior PCI, with the goal of achieving balance defined by a standardized mean difference in less than 0.10. In the propensity-matched cohort, normally distributed continuous variables were compared using the paired Student’s *t*-test, while non-normally distributed variables were analyzed using the Wilcoxon signed-rank test. No missing data were present, as all preoperative characteristics were required for propensity matching, and postoperative outcomes were readily available from patient records.

To assess potential multicollinearity, the variance inflation factor (VIF) was calculated for each variable in the propensity model. A VIF greater than 5 was considered indicative of significant collinearity between a given predictor and other variables in the model, warranting further evaluation [15].

A linear regression analysis was performed to evaluate the association between hospital costs and relevant clinical and procedural variables. The results of the regression were reported as beta coefficients (β) with corresponding standard errors (±SE). Only parameters with a *p* < 0.1 were included in the multivariable model.

All statistical tests were two-sided, with an alpha level of 0.05 considered statistically significant. Analyses were conducted using R (version 4.4.0, R Project for Statistical Computing, Vienna, Austria) within the RStudio 2024.04.1 environment. The data supporting this study’s findings are available upon reasonable request from the corresponding author, subject to institutional approval. This study adhered to the Strengthening the Reporting of Observational Studies in Epidemiology (STROBE) guidelines [16].

## 3. Results

Between 2018 and 2023, a total of 637 SAVR procedures were performed at our center. Of these, 303 patients underwent minimally invasive isolated SAVR, including 241 via PS and 62 via RAT. Following PSM, 62 balanced pairs were obtained, with only diabetes and hypertension showing a SMD slightly over 0.10. The covariate balance is illustrated in the Love plot (Figure S1), and no issues with multicollinearity were identified, as all VIF values were below 2 in the propensity model (Table S1). In the matched cohort, the median follow-up duration was 1.64 [0.73–4.09] years. Table 1 displays the baseline characteristics of the matched population, while Table S2 presents those of the unmatched population.

### 3.1. Clinical Outcomes

In the matched cohort, there was a significant difference in valve choice depending on the group (*p* < 0.001). Moreover, patients undergoing PS required significantly more intraoperative and postoperative transfusions than those undergoing RAT (43.5% vs. 16.1%, *p* = 0.002 and 53.2% vs. 25.8%, *p* = 0.003, respectively). Additionally, total intensive care unit (ICU) stay was longer in the PS group compared to the RAT group (52.2 h [44.7, 87.7] vs. 45.7 h [28.4, 69.2], *p* = 0.007). However, there were no other significant differences between the groups including bleeding requiring surgery, echocardiographic parameters, length of hospital stay, or 30-day mortality. The perioperative outcomes for the matched and unmatched population are presented in Table 2 and Table S3, respectively.

### 3.2. Hospital Cost Analysis

Matched hospital cost outcomes are summarized in Table 3. Among direct cost components, only blood bank expenses differed significantly between PS and RAT, with higher costs in the PS group ($240 [0–1090.75] vs. $128 [0–277.5], *p* = 0.010), reflecting the increased need for perioperative blood transfusions. No other significant differences were observed in direct costs between the two groups. However, the overall financial burden was higher for PS, with significantly greater average direct, indirect, and total costs compared to RAT ($40,915.62 ± 10,243.13 vs. $37,402.22 ± 7962.08, *p* = 0.038; $24,489.87 ± 5711.73 vs. $22,473.52 ± 4906.97, *p* = 0.040; $65,405.48 ± 15,717.97 vs. $59,875.73 ± 12,569.52, *p* = 0.035, respectively).

Multivariable linear regression analysis, conducted on the overall population, identified intraoperative (*p* = 0.024) and postoperative transfusions (*p* = 0.012), total ICU length of stay (*p* = 0.005), and hospital length of stay (*p* < 0.001) as significant predictors of hospital costs (Table 4). Given the significantly longer total ICU stay in the PS group, this factor contributed to the higher hospital costs observed in comparison to the RAT group.

## 4. Discussion

In this retrospective study using a PSM cohort, we compared SAVR outcomes and hospital costs between RAT and PS approaches. The main findings of this study were as follows: (1) significant differences were found in intraoperative and postoperative transfusions, as well as in median ICU hours; (2) average direct, indirect, and total hospital costs were significantly higher for PS, with significant blood bank cost differences favoring RAT; (3) multivariable linear regression showed that intraoperative and postoperative transfusions, ICU, and hospital length of stay were significantly associated with hospital costs in the overall population, suggesting a possible in-hospital cost benefit of RAT compared to PS considering the clinical outcomes related to each group.

Many studies have found no significant differences in mid- and long-term mortality between SAVR performed via PS or RAT. A PSM analysis comparing 202 patient pairs reported similar 30-day and 1-year mortality rates between the two approaches [6]. A meta-analysis of nine studies involving 2926 patients also found no difference in in-hospital mortality but noted that RAT was associated with shorter hospital stays and reduced mechanical ventilation duration [10]. Although RAT appeared to offer better short-term recovery, long-term survival data were lacking. Similarly, a more recent meta-analysis reported that RAT was associated with shorter hospitalization and lower rates of bleeding, transfusion, and acute kidney injury, although it may involve modestly prolonged cardiopulmonary bypass times and increased incision-related pain [17]. In line with these findings, Lim et al. observed that RAT resulted in fewer blood transfusions, shorter hospital stays, and a higher rate of immediate post-operative extubation [5]. Consistent with the mentioned studies, we observed lower transfusion rates and a shorter total ICU stay for RAT compared to PS. Of note, although the difference in ICU stay between groups reached statistical significance, the difference was approximately 6 h, which is modest from a clinical standpoint. The precise mechanism underlying this finding cannot be determined and would remain largely speculative. Mobility and drainage management were comparable between groups, as ICU care protocols were identical and not influenced by surgical approach. Postoperative pain itself, as opposed to differences in analgesic management, and variations in bleeding or transfusion requirements may represent plausible contributors; however, the data necessary to formally assess these hypotheses were not available. Since hospital cost analysis was the primary focus of this study, only perioperative outcomes were assessed in this study.

Even regarding the lower rates of transfusions in RAT compared to PS, the possible mechanisms remain speculative. It may be attributable to several complementary mechanisms, with sternal sparing being the most physiologically compelling [6,10]. In general, median or partial sternotomy necessitates division of the sternum, exposing highly vascular bone marrow that represents one of the most hemorrhagic steps of the procedure, requiring extensive hemostatic maneuvers. By completely preserving the sternum, RAT eliminates this source of blood loss entirely. Additionally, RAT avoids the broad soft-tissue dissection and retrosternal raw surfaces inherent to sternotomy, further limiting postoperative mediastinal ooze.

On the other hand, the review by Hassan and colleagues reported that PS patients observed a significantly shorter length of stay, cardiopulmonary bypass and aortic cross-clamp time, and lower transfusion rates compared to RAT [11]. However, these findings were based on studies comparing each minimally invasive technique to MS rather than a direct comparison. A recent multi-center PSM study found that hospital mortality at 30 days was significantly lower in the PS group [18]. Five independent predictors of 30-day mortality were identified, with the RAT approach being the strongest (odds ratio: 4.24, *p* = 0.002). The RAT group had longer operative times, higher conversion rates to MS, and increased ICU and hospital stays. No significant differences were found in CPB time, cross-clamp time, or overall hospital stay. As elegantly discussed by Balmforth et al., high-quality evidence comparing RAT and PS for minimally invasive SAVR is limited, as no randomized controlled trials have been conducted [19]. Conflicting data exist regarding early mortality and postoperative outcomes between these two minimally invasive SAVR approaches, also considering the different expertise in the techniques, and how they are surgically performed by each surgeon or center.

The review by Hassan et al. is currently the only study performing a cost–benefit analysis comparison between RAT and PS [11]. This analysis was based on institutional data and estimates from the literature, focusing on intraoperative expenses. The study found that, compared to conventional SAVR, RAT incurred in higher additional cost per case compared to PS. The higher costs associated with RAT were primarily due to the use of disposable catheters and cannulas for peripheral CPB, myocardial protection, and cardiac venting. This study is limited by the lack of a direct comparison, relying on indirect data and estimated financial figures rather than actual cost data. The cost of minimally invasive SAVR has been evaluated in only a few studies and is often perceived as higher due to longer operative times and the need for specialized equipment. However, no direct comparison between RAT and PS was conducted. Prior research on cost-effectiveness has yielded inconclusive results. An analysis of registry data on patients undergoing isolated primary AVR found that procedures performed via a RAT were associated with lower hospital costs compared to sternotomy-based approaches, without significant differences in clinical outcomes [20]. The primary drivers of these cost savings were shorter hospital stays and decreased reliance on blood transfusions. Ghanta et al. reported that total hospital costs for minimally invasive SAVR were 5% lower than for conventional AVR, largely due to earlier discharge and reduced blood product use [12]. Older single-center studies have also documented cost reductions for minimally invasive SAVR [21,22]. Variable outcomes were reported by the Mini-Stern trial [23]. The economic analysis showed that PS had higher costs than MS, with an additional cost of £1714 per patient in the first year post-surgery, though this was not statistically significant. After adjusting for baseline characteristics, PS was found to be more costly and less effective. Sensitivity analyses largely confirmed this, except in the complete case analysis, which suggested lower costs but slightly worse outcomes. However, this analysis was limited by a small sample size of 90 patients and only assessed costs within the first year, without long-term follow-up.

The current study has shown that the PS group had significantly higher blood bank expenses than the RAT group due to increased perioperative transfusions. While other direct costs did not differ significantly, overall costs were higher in the PS group, including direct, indirect, and total hospital costs. At multivariable regression analysis RAT (as grouping variable) did not retain independent significance in the multivariable regression model, suggesting that its effect on hospital costs is mediated through associated clinical outcomes only. Intraoperative and postoperative transfusions, ICU stay, and hospital stay were identified as significant predictors of hospital costs in the overall population. Postoperative care was delivered by the same ICU and step-down teams across both cohorts, minimizing variability in management. Accordingly, our interpretation is that the surgical approach per se does not directly determine hospital costs; rather, costs are influenced by the downstream clinical effects associated with the approach. The significantly longer ICU stay in the PS group may explain its higher overall costs compared to RAT. Indeed, it has been previously shown for SAVR that ICU stay may have the most important impact on costs [24]. However, in the present study, a rigorous testing linking the type of surgery with hospital costs requires assumptions that cannot be reliably satisfied in a retrospective observational design and remain hypothesis-generating.

Finally, it is important to acknowledge that the findings of the present study reflect the specific experience of our institution and should not be extrapolated uncritically to other clinical or economic contexts. Healthcare financing is inherently heterogeneous: reimbursement models, procurement costs, staffing structures, and negotiated agreements with device manufacturers vary considerably not only across countries, but also between hospitals within the same national system. The cost dynamics observed in our cohort are therefore shaped, at least in part, by the financial mechanisms unique to our institution, and a similar analysis conducted in a different setting may yield meaningfully different results.

### Limitations

This study is subject to limitations, including its retrospective, single-center design. Additionally, the small sample size following propensity score matching, largely due to the limited number of patients in the pre-matched cohort, along with the low event rate, may reduce statistical power and limit the generalizability of the results. Although PSM helps create balanced groups, the possibility of bias due to unmeasured or unidentified confounders remains, as is often the case in observational studies. Residual confounding due to surgeon selection and procedural indication could not be eliminated. Postoperative total drainage volume was unavailable, limiting a comprehensive evaluation of postoperative bleeding. Another limitation of this analysis is that it focuses solely on hospital costs, without incorporating a more comprehensive cost-effectiveness analysis that considers factors such as post-discharge expenses, rehabilitation, and long-term healthcare utilization. A further limitation of this study is the absence of itemized surgical cost data, precluding a granular analysis of the individual cost contributions of prosthesis type, cannulation strategy, and extracorporeal circuit materials. This narrower scope may not fully capture the overall economic impact of the procedures, highlighting the need for future studies with a more extensive cost evaluation. Indeed, to validate these findings, future research should focus on larger, prospectively collected cohorts. These findings reflect our institutional experience and practice patterns, and should be interpreted as hypothesis-generating rather than conclusive.

## 5. Conclusions

The present study showed that PS and RAT for SAVR had comparable clinical outcomes, with differences observed only in the number of transfusions and ICU stay, both favoring RAT. Given the significant perioperative differences and regression analysis results, the cost advantage of RAT is likely mediated through its impact on these perioperative outcomes.

## Figures and Tables

**Table 1:** Matched patient’s baseline characteristics.

Baseline Characteristics	PS, n = 62	RAT, n = 62	SMD
Males, n (%)	40 (64.5)	38 (61.3)	−0.066
Age, years, mean (SD)	67.37 (8.87)	67.69 (7.38)	0.044
BMI, kg/m^2^, median [IQR]	27.56 [25.71, 30.42]	27.80 [24.69, 31.33]	0.043
NYHA III-IV, n (%)	3 (4.8)	2 (3.2)	−0.091
Urgent status, n (%)	0 (0.0)	0 (0.0)	0.000
STS score, median [IQR]	0.94 [0.67, 1.21]	0.82 [0.60, 1.30]	0.046
COPD (%)	12 (19.4)	13 (21.0)	0.040
History of smoke (%)	24 (38.7)	26 (41.9)	0.065
Hemoglobin, g/dL, mean (SD)	13.8 (1.6)	13.8 (1.3)	0.066
Creatinine, mg/dL, median [IQR]	0.90 [0.80, 1.00]	0.90 [0.80, 1.10]	0.038
Dialysis, n (%)	0 (0.0)	0 (0.0)	0.000
Diabetes, n (%)	14 (22.6)	11 (17.7)	−0.127
Hypertension, n (%)	45 (72.6)	40 (64.5)	−0.169
Prior CVA, n (%)	2 (3.2)	3 (4.8)	0.075
PAD, n (%)	3 (4.8)	2 (3.2)	−0.091
Liver disease, n (%)	2 (3.2)	2 (3.2)	0.000
Prior PCI, n (%)	4 (6.5)	5 (8.1)	0.059
PM and/or ICD, n (%)	3 (4.8)	2 (3.2)	−0.091
Atrial fibrillation, n (%)	7 (11.3)	6 (9.7)	−0.055
Ejection fraction, %, median [IQR]	65.00 [63.00, 70.00]	65.00 [60.75, 70.00]	−0.017

AVA = aortic valve area; BMI = body mass index; BSA = body surface area; COPD = chronic obstructive pulmonary disease; CVA = cerebrovascular accident; ICD = implantable cardioverter defibrillator; IQR = interquartile range; MI = myocardial infarction; NYHA = New York Heart Association; PAD = peripheral artery disease; PCI = percutaneous coronary intervention; PG = pressure gradient; PM = pacemaker; PS = partial sternotomy; RAT = right anterior thoracotomy; SD = standard deviation; SMD = standardized mean difference; STS = Society of Thoracic Surgeons.

**Table 2:** Matched perioperative outcomes.

Outcomes	PS, n = 62	RAT, n = 62	*p*-Value
Intraoperative transfusions, n (%)	27 (43.5)	10 (16.1)	**0.002**
CPB time, min, median [IQR]	101.00 [91.00, 113.50]	102.50 [85.25, 119.75]	0.770
CXC time, min, median [IQR]	71.50 [64.75, 81.75]	74.00 [58.50, 92.00]	0.818
Type of valve, n (%)			**<0.001**
Stented	57 (91.9)	36 (58.1)	
Sutureless	4 (6.5)	26 (41.9)	
Mechanical	1 (1.6)	0 (0.0)	
Skin-to-skin time, hours, mean (SD)	3.73 (0.78)	3.84 (0.64)	0.514
Extubated in OR, n (%)	34 (54.8)	40 (64.5)	0.360
Total ventilation hours, median [IQR]	3.92 [2.74, 5.64]	3.62 [2.88, 5.22]	0.815
Total ICU hours, median [IQR]	52.23 [44.67, 87.73]	45.74 [28.38, 69.21]	**0.007**
CVA, n (%)	0 (0.0)	0 (0.0)	>0.999
DWI, n (%)	0 (0.0)	0 (0.0)	>0.999
Reop for valve dysfunction, n (%)	0 (0.0)	0 (0.0)	>0.999
Bleeding requiring surgery, n (%)	1 (1.6)	1 (1.6)	>0.999
Creatinine, mg/dL, median [IQR]	1.00 [0.80, 1.10]	1.10 [0.90, 1.20]	0.122
Postoperative dialysis, n (%)	0 (0.0)	0 (0.0)	>0.999
Myocardial infarction, n (%)	0 (0.0)	0 (0.0)	>0.999
New AF, n (%)	16 (25.8)	20 (32.3)	0.553
New PM/ICD = 1 (%)	4 (6.5)	3 (4.8)	>0.999
Postoperative transfusions, n (%)	33 (53.2)	16 (25.8)	**0.003**
Effective orifice area, cm^2^, median [IQR]	1.34 [1.10, 1.81]	1.38 [1.12, 1.69]	0.876
Mean gradient, mmHg, median [IQR]	10 [8,14]	12 [9,17]	0.204
Paravalvular leaks	0 (0.0)	0 (0.0)	>0.999
Length of stay, median [IQR]	4.00 [4.00, 6.00]	4.00 [4.00, 6.00]	0.706
30-day mortality, n (%)	1 (1.6)	0 (0.0)	>0.999

Bold means statistically significant at *p* < 0.05. AF = atrial fibrillation; CPB = cardiopulmonary bypass; CVA = cerebrovascular accident; CXC = cross clamp; DWI = deep wound infection; ICD = implantable cardioverter defibrillator; ICU = intensive care unit; IQR = interquartile range; OR = operating room; PM = pacemaker; PS = partial sternotomy; RAT = right anterior thoracotomy; SMD = standardized mean difference.

**Table 3:** Matched hospital cost analysis.

Hospital Costs	PS, n = 60	RAT, n = 60	*p*-Value
Nursing, $, median [IQR]	6159.50 [4867.00, 8300.75]	5127.00 [4244.50, 6617.25]	0.112
Surgical time, $, median [IQR]	6145.00 [5651.50, 6905.50]	6101.00 [5628.50, 6783.50]	0.910
Surgical implants, $, mean (SD)	11,365.37 (3231.90)	10,179.98 (4532.95)	0.102
Surgical other, $, median [IQR]	7304.50 [5750.25, 8685.75]	7007.50 [6331.50, 7873.25]	0.448
Cath Lab, $, median [IQR]	0.00 [0.00, 0.00]	0.00 [0.00, 0.00]	0.525
Pharmacology, $, median [IQR]	1987.50 [1586.75, 3452.00]	1909.50 [1549.25, 2170.25]	0.242
Blood bank, $, median [IQR]	240 [0–1090.75]	128 [0–277.5]	**0.010**
Radiology, $, median [IQR]	188.00 [153.00, 245.25]	186.00 [150.00, 271.75]	0.763
Cardiology, $, median [IQR]	114.00 [81.75, 197.00]	125.50 [67.00, 208.75]	0.821
Physical medicine, $, median [IQR]	145.00 [120.00, 179.00]	145.00 [119.00, 179.00]	0.746
Laboratory, $, median [IQR]	698.00 [605.25, 805.50]	700.50 [578.50, 851.50]	0.981
Respiratory therapy, $, median [IQR]	317.00 [0.00, 480.00]	106.50 [0.00, 411.00]	0.221
Physician fees, $, median [IQR]	3355.00 [1400.00, 3965.00]	3355.00 [1058.75, 3965.00]	0.868
Average direct cost, $, mean (SD)	40,915.62 (10,243.13)	37,402.22 (7962.08)	**0.038**
Average indirect cost, $, mean (SD)	24,489.87 (5711.73)	22,473.52 (4906.97)	**0.040**
Average total cost, $, mean (SD)	65,405.48 (15,717.97)	59,875.73 (12,569.52)	**0.035**
Total direct cost, $	2,457,937	2,244,133	
Total indirect cost, $	1,469,392	1,348,411	
Total cost, $	3,924,329	3,592,544	

Bold means statistically significant at *p* < 0.05. IQR = interquartile range; PS = partial sternotomy; RAT = right anterior thoracotomy; SD = standard deviation.

**Table 4:** Matched hospital cost analysis regression.

Hospital Cost Regression	Univariable Estimate (β) ± SE, *p*-Value	Multivariable Estimate (β) ± SE, *p*-Value
RAT (ref: PS)	−5530 ± 2598, **0.035**	−109.0 ± 2057.3, 0.958
Hemoglobin	−951 ± 922.4, 0.305	-
STS score	3959 ± 2165, 0.070	−1772.1 ± 1826.4, 0.336
Skin-to-skin time	5177 ± 2167, **0.020**	2263.7 ± 1367.8, 0.103
Intraoperative transfusions	9197 ± 2787, **0.001**	6267.3 ± 2718.4, **0.024**
Total ICU hours	290.83 ± 29.49, **<0.001**	113.8 ± 39.4, **0.005**
Postoperative transfusions	8940 ± 2574, **<0.001**	6424.4 ± 2489.1, **0.012**
Length of hospital stay	3447.4 ± 613.9, **<0.001**	4049.2 ± 804.6, **<0.001**

Bold means statistically significant at *p* < 0.05. ICU = intensive care unit; PS = partial sternotomy; RAT = right anterior thoracotomy; SE = standard error; STS = Society of Thoracic Surgeons.

## Data Availability

The data that support the findings of this study are available upon reasonable request to the corresponding author, pending institutional approval.

## Supplementary Materials

The following supporting information can be downloaded at: https://www.mdpi.com/article/10.3390/medicina62050856/s1. Figure S1: Love plot depicting the effectiveness of propensity score matching in balancing key covariates between groups; Table S1: Variance inflation factor of the propensity model; Table S2: Unmatched patients’ baseline characteristics; Table S3: Unmatched perioperative outcomes.

## Abbreviations

The following abbreviations are used in this manuscript:

ICU	Intensive care unit
MS	Median sternotomy
PS	Partial upper sternotomy
PSM	Propensity score match
RAT	Right anterior mini-thoracotomy
SAVR	Surgical aortic valve replacement
VIF	Variance inflation factor
