# Supplementary material for: Right Anterior Thoracotomy Versus Partial Sternotomy for Isolated Aortic Valve Replacement: A Propensity Analysis of Clinical Outcomes and Hospital Costs
Source: Medicina (Kaunas). 2026 Apr 30;62(5):856. doi: 10.3390/medicina62050856 (PMC13208599; doi:10.3390/medicina62050856)
Supplement: Supplementary file 1 [file medicina-62-00856-s001.zip › Supplementary Tables.pdf]

Supplementary Table S1 – Variance inflation factor of the propensity model

Hypertension	1.167045
Age	1.322281
Sex	1.49047
STS-PROM	1.941445
NYHA 3/4	1.02951
Ejection fraction	1.08752
Diabetes	1.056365
Prior PCI	1.042173
Smoke	1.050191
BMI	1.026884
COPD	1.157827
Urgency	1
Atrial fibrillation	1.048406
Liver disease	1.057699

Supplementary Table S2 – Unmatched patient's baseline characteristics

	PS<br>N=241	RAT<br>N=62	p-value	SMD
Males, n (%)	163 (67.6)	38 (61.3)	0.428	-0.1303
Age, years, mean (SD)	68.50 (8.52)	67.69 (7.38)	0.494	-0.1095
BMI, kg/m^2, median [IQR]	29.13 [25.82, 33.10]	27.80 [24.69, 31.33]	0.136	-0.2096
NYHA III-IV, n (%)	37 (15.4)	2 (3.2)	**0.010**	-0.6864
Urgent status, n (%)	3 (1.2)	0 (0.0)	>0.999	-0.1257
STS score, median [IQR]	1.04 [0.74, 1.42]	0.82 [0.60, 1.30]	**0.018**	-0.1858
COPD (%)	38 (15.8)	13 (21.0)	0.432	0.1277
History of smoke (%)	127 (52.7)	26 (41.9)	0.155	-0.2181
Hemoglobin, g/dL, mean (SD)	13.6 (1.6)	13.8 (1.3)	0.178	0.2030
Creatinine, mg/dL, median [IQR]	0.90 [0.80, 1.10]	0.90 [0.80, 1.10]	0.615	-0.1460
Dialysis, n (%)	0 (0.0)	0 (0.0)	>0.999	0.0000
Diabetes, n (%)	73 (30.3)	11 (17.7)	0.070	-0.3285
Hypertension, n (%)	185 (76.8)	40 (64.5)	0.071	-0.2560
Prior CVA, n (%)	11 (4.6)	3 (4.8)	>0.999	0.0128
PAD, n (%)	11 (4.6)	2 (3.2)	>0.999	-0.0758
Liver disease, n (%)	5 (2.1)	2 (3.2)	0.635	0.0652
Prior PCI, n (%)	38 (15.8)	5 (8.1)	0.178	-0.2829
PM and/or ICD, n (%)	9 (3.7)	2 (3.2)	>0.999	-0.0288
Atrial fibrillation, n (%)	24 (10.0)	6 (9.7)	>0.999	-0.0095
Ejection fraction, %, median [IQR]	63.00 [59.00, 68.00]	65.00 [60.75, 70.00]	**0.009**	0.5964

AVA = aortic valve area; BMI = body mass index; BSA = body surface area; COPD = chronic obstructive pulmonary disease; CVA = cerebrovascular accident; ICD = implantable cardioverter defibrillator; IQR = interquartile range; MI = myocardial infarction; NYHA = New York Heart Association; PAD = peripheral artery disease; PCI = percutaneous coronary intervention; PG = pressure gradient; PM = pacemaker; PS = partial sternotomy; RAT = right anterior thoracotomy; SD = standard deviation; SMD = standardized mean difference; STS = Society of Thoracic Surgeons.

Supplementary Table S3 – Unmatched perioperative outcomes

	PS<br>N=241	RAT<br>N=62	p-value
Intraoperative transfusions, n (%)	108 (44.8)	10 (16.1)	**<0.001**
CPB time, min, median [IQR]	101.00 [91.00, 115.00]	102.50 [85.25, 119.75]	0.959
CXC time, min, median [IQR]	73.00 [64.00, 82.00]	74.00 [58.50, 92.00]	0.822
Type of valve, n (%)			**<0.001**
Stented	229 (95.0)	36 (58.1)	
Sutureless	9 (3.7)	26 (41.9)	
Mechanical	3 (1.2)	0 (0.0)	
Skin-to-skin time, hours, mean (SD)	3.72 (0.68)	3.84 (0.64)	0.326
Extubated in OR, n (%)	100 (41.5)	40 (64.5)	**0.002**
Total ventilation hours, median [IQR]	4.11 [2.87, 6.60]	3.62 [2.88, 5.22]	0.377
Total ICU hours, median [IQR]	54.55 [46.57, 86.48]	45.74 [28.38, 69.21]	**<0.001**
CVA, n (%)	4 (1.7)	0 (0.0)	0.585
DWI, n (%)	0 (0.0)	0 (0.0)	>0.999
Reop for valve dysfunction, n (%)	0 (0.0)	0 (0.0)	>0.999
Bleeding requiring surgery, n (%)	2 (0.8)	1 (1.6)	0.498
Creatinine, mg/dL, median [IQR]	1.10 [0.90, 1.30]	1.10 [0.90, 1.20]	0.453
Postoperative dialysis, n (%)	1 (0.4)	0 (0.0)	>0.999
Myocardial infarction, n (%)	0 (0.0)	0 (0.0)	>0.999
New AF, n (%)	69 (28.6)	20 (32.3)	0.687
New PM/ICD = 1 (%)	13 (5.4)	3 (4.8)	>0.999
Postoperative transfusions, n (%)	131 (54.4)	16 (25.8)	**<0.001**
Effective orifice area, cm^2, median [IQR]	1.51 [1.11, 1.80]	1.38 [1.12, 1.69]	0.371
Mean gradient, mmHg, median [IQR]	11 [9, 14]	12 [9, 17]	0.883
Paravalvular leaks	2 (0.8)	0 (0.0)	>0.999
Length of stay, median [IQR]	5.00 [4.00, 6.00]	4.00 [4.00, 6.00]	**0.007**
30-day mortality, n (%)	1 (0.4)	0 (0.0)	>0.999

AF = atrial fibrillation; CPB = cardiopulmonary bypass; CVA = cerebrovascular accident; CXC = cross clamp; DWI = deep wound infection; ICD = implantable cardioverter defibrillator; ICU = intensive care unit; IQR = interquartile range; OR = operating room; PM = pacemaker; PS = partial sternotomy; RAT = right anterior thoracotomy; SMD = standardized mean difference
