# Supplementary figures and images for: Right Anterior Thoracotomy Versus Partial Sternotomy for Isolated Aortic Valve Replacement: A Propensity Analysis of Clinical Outcomes and Hospital Costs
Source: Medicina (Kaunas). 2026 Apr 30;62(5):856. doi: 10.3390/medicina62050856 (PMC13208599; doi:10.3390/medicina62050856)

# Covariate Balance

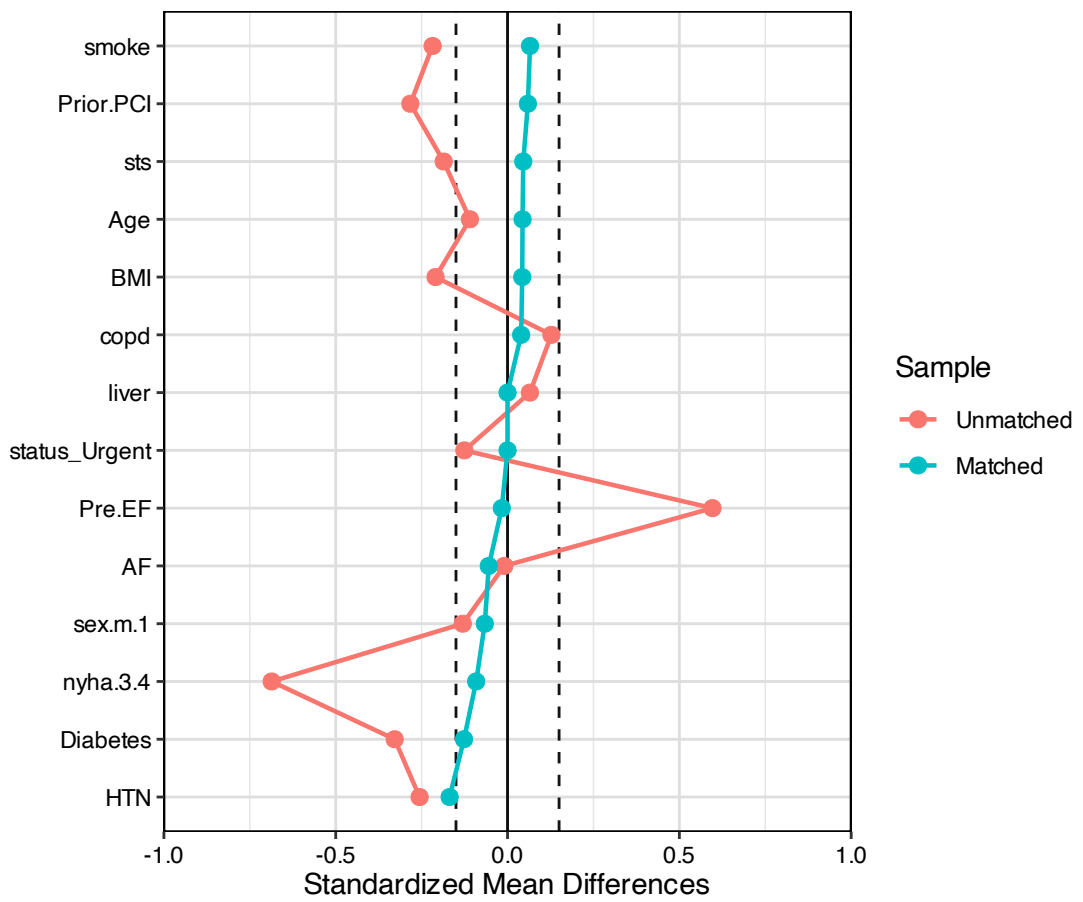

Supplement: Supplementary file 1 [file medicina-62-00856-s001.zip › Supplementary Figure.pdf]
